# Identification of potential targets of the curcumin analog CCA-1.1 for glioblastoma treatment : integrated computational analysis and in vitro study

**DOI:** 10.1038/s41598-022-18348-9

**Published:** 2022-08-17

**Authors:** Adam Hermawan, Febri Wulandari, Naufa Hanif, Rohmad Yudi Utomo, Riris Istighfari Jenie, Muthi Ikawati, Ahmad Syauqy Tafrihani

**Affiliations:** 1 Faculty of Pharmacy, Cancer Chemoprevention Research Center, Universitas Gadjah Mada Sekip Utara II, Yogyakarta, 55281 Indonesia; 2 Laboratory of Macromolecular Engineering, Department of Pharmaceutical Chemistry, Faculty of Pharmacy, Universitas Gadjah Mada Sekip Utara II, Yogyakarta, 55281 Indonesia; 3 Laboratory of Medicinal Chemistry, Department of Pharmaceutical Chemistry, Faculty of Pharmacy, Universitas Gadjah Mada Sekip Utara II, Yogyakarta, 55281 Indonesia

**Keywords:** Computational biology and bioinformatics, Data mining, CNS cancer, Drug screening, Target identification

## Abstract

The treatment of glioblastoma multiforme (GBM) is challenging owing to its localization in the brain, the limited capacity of brain cells to repair, resistance to conventional therapy, and its aggressiveness. Curcumin has anticancer activity against aggressive cancers, such as leukemia, and GBM; however, its application is limited by its low solubility and bioavailability. Chemoprevention curcumin analog 1.1 (CCA-1.1), a curcumin analog, has better solubility and stability than those of curcumin. In this study, we explored potential targets of CCA-1.1 in GBM (PTCGs) by an integrated computational analysis and in vitro study. Predicted targets of CCA-1.1 obtained using various databases were subjected to comprehensive downstream analyses, including functional annotation, disease and drug association analyses, protein–protein interaction network analyses, analyses of genetic alterations, expression, and associations with survival and immune cell infiltration. Our integrative bioinformatics analysis revealed four candidate targets of CCA-1.1 in GBM: TP53, EGFR, AKT1, and CASP3. In addition to targeting specific proteins with regulatory effects in GBM, CCA-1.1 has the capacity to modulate the immunological milieu. Cytotoxicity of CCA-1.1 was lower than TMZ with an IC50 value of 9.8 μM compared to TMZ with an IC50 of 40 μM. mRNA sequencing revealed EGFR transcript variant 8 was upregulated, whereas EGFRvIII was downregulated in U87 cells after treatment with CCA-1.1. Furthermore, a molecular docking analysis suggested that CCA-1.1 inhibits EGFR with various mutations in GBM, which was confirmed using molecular dynamics simulation, wherein the binding between CCA-1.1 with the mutant EGFR L861Q was stable. For successful clinical translation, the effects of CCA-1.1 need to be confirmed in laboratory studies and clinical trials.

## Introduction

Glioblastoma multiforme (GBM) is one of the most prevalent and aggressive brain tumors^1^. GBM arises from astrocytes that support nerve cells and invade nearby brain cells^2^. It can affect children; however, it is more common in adults aged 40–75 years^3^. Standard therapies for GBM currently include surgery followed by radiotherapy and chemotherapy. The standard chemotherapy is temozolomide, which is administered during radiation therapy^4^. Targeted chemotherapy drugs, such as lomustine (chemotherapy) and bevacizumab (anti-angiogenesis), are also administered in advanced GBM^5^. The treatment of GBM is challenging owing to its localization in the brain, the limited capacity of brain cells to repair, resistance to conventional therapy, and its aggressiveness^3^.

Immune cells participate in the disease progression of liver fibrosis^6^ and GBM^7^. The importance of interactions between tumors and their microenvironment in disease progression, including GBM progression, is now well-established^8^. The tumor microenvironment involves chronic inflammation, involving fibroblasts, pericytes, and immune cells^9^. However, the immune microenvironment of GBM is extremely immunosuppressive because of the lack of immune effector cell types and tumor-infiltrating lymphocytes^7^, making it challenging to target immune cells. The measurement of immune cell infiltration^10^ in GBM is an important tool for predicting clinical outcomes^11,12^, as a prognostic marker and a predictor of therapeutic outcomes.

**Figure 1:**
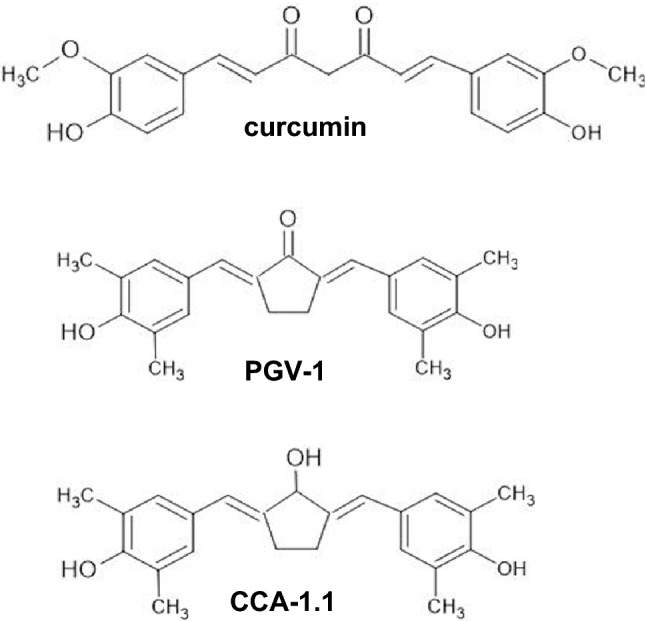
Chemical structures of curcumin, PGV-1, and CCA-1.1.

To address the hurdles limiting effective GBM treatment, we explored new therapeutic compounds related to curcumin (Fig. 1), which has anticancer activity against various aggressive cancers, such as colon cancer, leukemia, and GBM^13^. Curcumin has been shown to increase the sensitivity of GBM cells to cisplatin, etoposide, camptothecin, and doxorubicin^14^. Curcumin exerts therapeutic effects in GBM via multiple pathways, including the suppression of AKT/mTOR and activation of ERK1/2 pathways in human malignant glioma U87-MG and U373-MG with PTEN mutations^15^. Furthermore, the effect of curcumin on the ERK pathway promotes the activation of p21, as observed by Choi et al. ^16^ The curcumin-induced inhibition of GBM cell proliferation and chemoresistance is mediated by AP-1 and NF-κB^14^. An in vivo study by Perry et al. (2010) revealed that curcumin affects glioblastoma growth and angiogenesis in mice with U87 glioma xenografts^17^. In addition, Facina et al. (2021) demonstrated the anticarcinogenic effect of curcumin alone, and in combination with piperin, in bisphenol A-induced carcinogenesis in gerbil prostates^18^. Moreover, in vitro and in silico studies by Liang et al. (2021) successfully synthesized curcumin and its analog and suggested their potential as EGFR inhibitors, in which curcumin and its analog regulate the expression of EGFR^19^. A recent study has demonstrated that the curcumin analog dimethoxycurcumin promotes apoptosis, autophagy, and ROS production and suppresses cell viability in human gliomas^20^.

Natural products such as *Tripterygium wilfordii*^21^ and *Ganoderma*^22^ have immunomodulatory effects by inhibiting the expression of pro-inflammatory cytokines, and the production of other cytokines and antibodies. Curcumin exerts anticancer activity, in part, by modulating the immune system. A previous study has shown that curcumin increases the efficacy of immunotherapy in melanoma cells^23^. Additionally, curcumin is a promising immunotherapy for GBM^24^. A previous study showed that curcumin can be used in immunotherapy by decreasing the expression of immune checkpoint ligands and restoring the CD8 + cell function in head and neck cancer cells^25^. As discussed in a recent review, curcumin promotes immune function to eliminate cancer cells via several mechanisms^26^, however, its application is limited by its low solubility and bioavailability^27^.

Chemoprevention curcumin analog 1.1 (CCA-1.1), shown in Fig. 1, is a curcumin analog, with a substitution of the ketone group in the cyclopentane structure of PGV-1 (Fig. 1), a former analog, with a hydroxyl group; it has better solubility and stability than those of curcumin and PGV-1^28^. CCA-1.1 also exhibits better anticancer activity than that of PGV-1 in several cancer cells, including luminal A MCF-7, HER2-positive HCC1954, triple-negative 4T1 breast cancer cells, K562 leukemic cells, Caco2, and WiDr colon cancer cells^28^. CCA-1.1 is able to induce cell cycle arrest and senescence^29^, increase the cytotoxicity of doxorubicin^30^, and hamper migration in T47D, estrogen-positive breast cancer cells^31^ and in WiDr colon cancer cells^32^. CCA-1.1 also inhibits the migration of triple-negative and HER2-positive breast cancer cells^33^ and induces mitotic arrest in triple-negative breast cancer^34^. Bioinformatics studies have explored the target genes of CCA-1.1 in colon cancer^35^ and triple-negative breast cancer cells^36^; however, similar analyses have not been performed for GBM.

**Figure 2:**
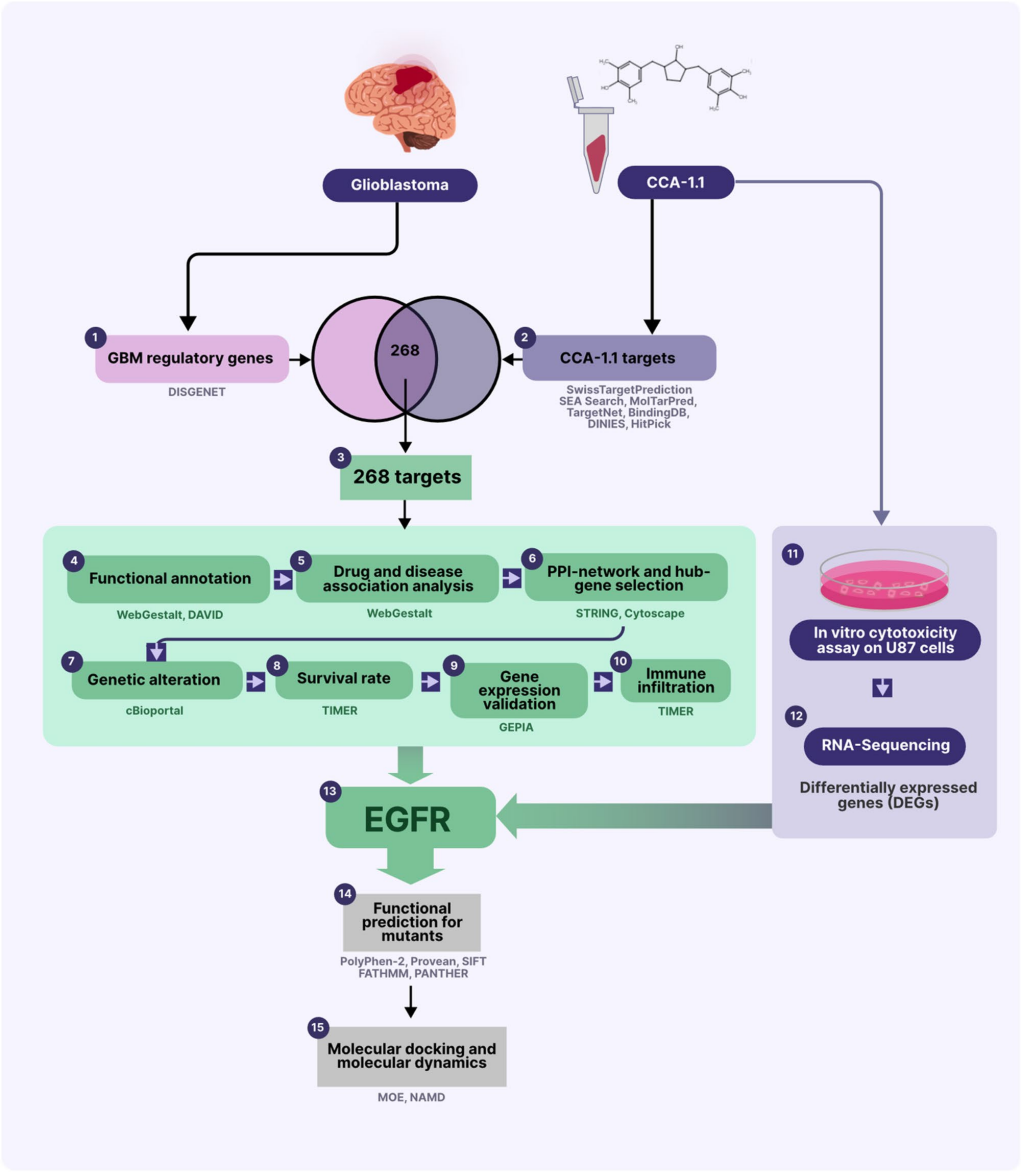
Work flow of the study.

In this study, we explored potential targets of CCA-1.1 in GBM (PTCG) by an integrated computational analysis and in vitro study (Fig. 2). Targets of CCA-1.1 were predicted from public databases and further analyzed for the selection of candidates. Our results indicate that CCA-1.1 not only targets certain regulatory genes in GBM but also modulates the immune environment.

## Methods

### Data mining

Protein targets of CCA-1.1 were predicted from several databases, including SwissTargetPrediction (http://www.swisstargetprediction.ch)^37^, SEA Search (https://sea.bkslab.org)^38^, MolTarPred (https://moltarpred.marseille.inserm.fr)^39^, TargetNet (http://targetnet.scbdd.com)^40^, BindingDB (https://www.bindingdb.org)^41^, DINIES (https://www.genome.jp/tools/dinies/)^42^, and HitPick (http://mips.helmholtz-muenchen.de/proj/hitpick)^43^ using the default settings for each database, as described previously^35^. Regulatory genes associated with GBM were obtained by searching against DISGENET https://www.disgenet.org^44^ with the keyword human glioblastoma and default settings for the database.

### Functional annotation

Functional annotation of the PTCGs, including Gene Ontology and KEGG pathway enrichment analyses, was conducted using WebGestalt (http://www.webgestalt.org/)^45^ and DAVID version 6.8 (https://david.ncifcrf.gov)^46^, respectively. Briefly, the PTCGs were submitted to WebGestalt or DAVID as gene symbols and analyzed under default settings. FDR < 0.05 was selected as the cut-off value for the Gene Ontology analysis, and *p* < 0.05 was the threshold for KEGG pathway enrichment.

### Disease- and drug-gene association analyses

Disease–gene and drug–gene associations were analyzed using WebGestalt (http://www.webgestalt.org/)^45^. Briefly, for disease–gene associations, PTCGs were submitted for an Over-Representation Analysis (ORA) using WebGestalt, selecting OMIM as the disease and functional database. For drug–gene associations, PTCGs were submitted for an ORA using WebGestalt with the DrugBank database. FDR < 0.05 was selected as the cut-off value.

### Protein–protein interaction network construction and hub gene selection

A protein–protein interaction network for PTCG was constructed using STRING version 11.5 (https://string-db.org)^47^, with several parameters, including a confidence score of 0.4, *Homo sapiens*, and interaction between submitted protein symbols only. Hub genes, the genes with the top 10 degree scores were retained using the CytoHubba plugin of Cytoscape^48^ based on the degree score, as described previously^49^.

### Analysis of genetic alterations in hub genes

Genetic alterations in hub genes were analyzed using cBioportal (https://www.cbioportal.org)^50,51^. Briefly, hub genes were submitted as gene symbols to search for alterations reported in studies of GBM. Further analyses were performed, including visualization using OncoPrint and mutation plots as well as analyses of copy number alterations and related pathways.

### Expression of hub genes across glioblastoma samples

The expression levels of hub genes in GBM and normal tissues were compared using Gene Expression Profiling Interactive Analysis (GEPIA), (http://gepia.cancer-pku.cn/index.html)^52^. Briefly, gene symbols were submitted to GEPIA with the following parameter settings: GBM datasets, |Log2FC| cutoff = 2, *p* < 0.01, Jitter size of 0.4, and match TCGA normal and GTEx data.

### Survival analysis for hub genes

To evaluate the prognostic value of the hub genes, Kaplan–Meier survival curves were generated using TIMER 2.0 (http://timer.cistrome.org)^53,54^, applying the median cutoff and the glioblastoma multiforme (GBM) dataset.

### Correlations between hub genes and immune cell infiltration

Correlations between the expression levels of selected hub genes and immune cell infiltration were analyzed using TIMER 2.0 (http://timer.cistrome.org)^53,54^, using default settings, as described previously.

### Cells

U87 cells were kindly given by Dr. Muhammad Hasan Bashari, MD., Faculty of Medicine, Universitas Padjajaran, Bandung. The U87 cells were cultured in RPMI medium, containing 10% of fetal bovine serum (FBS, Gibco), 1% of penicillin–streptomycin (Gibco), and maintained in 5% of CO2 incubator. For the cytotoxicity assay, the U87 cells (3,000 cells/ well) were seeded in a 96-well-plate and incubated for 24 h prior to treatment of CCA-1.1, temozolomide (TMZ, purchased from Sigma), or DMSO for the following 72 h. TMZ was used as a control as TMZ is the first choice for GBM treatment^55^. DMSO was used as a co-solvent of CCA-1.1, and TMZ, and as a control at a maximum concentration of 1% (v/v). At the end of incubation, an MTT solution was added and incubated for 3 h prior to addition of 10% of SDS solution. Cell viability was calculated as previously described^56^. The IC50 value was calculated with GraphPad Prism 5.0 using non-linear regression (curve fit): log (agonist) vs. normalized response-variable slope.

### RNA sequencing

U87 cells were seeded, incubated, and treated with CCA-1.1 for 72 h. RNA isolation was performed using Bioline—Isolate II RNA Mini Kit, as per manufacturer’s instruction. Total RNA was then processed for mRNA enrichment, double-stranded cDNA synthesis, repair ends and addition of A overhang and A adaptor, fragment selection and PCR amplification, library quality testing, and next generation sequencing using Illumina HiSeq4000 from HiSeq-X sequencing technology. The qualities of the cleaned reads were assessed using FastQC version 0.11.9 (https://github.com/s-andrews/FastQC), and the reports were compiled using MultiQC version 1.1 (https://multiqc.info). The transcripts were quantified using the pseudo-alignment method employing Kallisto version 0.461^57^ with the human genome as a reference (GRCh38.p14). Differential expressed genes (DEGs) analysis was performed using EdgeR version 3.34.0^58^ using parameters such as ILogFCI > 1 and a *p* value < 0.05.

### Functional predictions for mutants

The effects of mutations on EGFR protein function were evaluated using several databases (with default parameters settings), including PolyPhen-2 (http://genetics.bwh.harvard.edu/pph2/dokuwiki/start)^59,60^, Provean (http://provean.jcvi.org/index.php)^61^, SIFT (https://sift.bii.a-star.edu.sg)^62^, FATHMM (http://fathmm.biocompute.org.uk)^60^, and PANTHER (http://www.pantherdb.org)^63^. PolyPhen-2 settings were as follows: batch query input; code: 3NJP; HumDiv & HumVar classifier; canonical transcripts; missense annotations; GRCh37/hg19 genome assembly. A higher score indicated a more deleterious effect on protein function. The Provean analysis was conducted using default settings of for Human Batch Protein Prediction, in which prediction scores of less than −2.5 indicate that a mutation is deleterious. SIFT was conducted using the following parameters: database UniProt-SwissProt + TrEMBL 2010_09; median conservation of sequences: 3.00; identical query threshold: 90%. A prediction score of five indicates “tolerated.” The FATHMM analysis was performed using the following parameters: cancer-relativity inherited disease; weighted prediction; phenotype association, disease ontology. A prediction score of less than −1.5 indicates a “damaging” mutation. The coding SNP that impacted protein function was predicted using PANTHER with the following interpretations of the probability of deleterious effect (Pdel): "probably damaging" (time > 450 my, corresponding to a false positive rate of ~ 0.2 as tested using HumVar), "possibly damaging" (450 my > time > 200 my, corresponding to a false positive rate of ~ 0.4), and "probably benign" (time < 200 my). Predictions were performed by comparing the mutant to wild-type EGFR (PDB ID: 3NJP).

### Molecular docking

The binding properties of curcumin and its analogs (PGV-1 and CCA-1.1) against EGFR and its mutant forms were predicted by a molecular docking analysis. Before performing the simulations, a template of the EGFR structure (UniProt code P00533) was retrieved from AlphaFold (https://alphafold.ebi.ac.uk/)^64^. The structures of mutant EGFR (E709K, T263P, V774M, and L861Q) were manually predicted using the MOE 2010 software, using the default step preparation. Due to the unknown binding site of each compound, the sitefinder in MOE was used to create a dummy site as the possible cavity for docking simulation. MOE 2010 (licensed from the Faculty of Pharmacy UGM) was also used for docking simulations, and the visualization of interactions. PGV-1 and CCA-1.1 structures were drawn using Marvin Sketch, and the curcumin structure was downloaded from PubChem. These structures were then subjected to conformational searches and energy minimization by MOE using the Energy Minimize Menu. For the docking simulation settings, London dG was used for both Rescoring 1 and Rescoring 2. Triangle Matcher was used for the score function and placement setting, and Forcefield was used to refine the docking results from 30 retained poses, as described previously^49^. The conformation with the lowest binding interaction between the ligand and receptor was determined.

### Molecular dynamics simulation

The results of molecular docking were validated using molecular dynamics (MD) simulation. As the representative, we chose the binding pocket of EGFR L861Q in complex with curcumin, PGV-1, and CCA-1.1. The MD simulation was completed in NAMD 2.14^65^ and visualized using VMD 1.9.4^66^. Parameterization of the proteins and ligands was prepared using CHARMM36 and CGenFF, available in the CHARMM-GUI web server^67^. For the solvation and neutralization steps, a cubic water box with 20-Å padding was added followed by K + and Cl^−^ ion addition. For equilibration, the complex was minimized for 70 ps and simulated for 1 ns. Further, a 1-ns simulation (NPT ensemble, pressure 1 atm, and temperature 303 K) was conducted to finalize the MD simulation process. The visualization and trajectories of the MD results were analyzed using root-mean-square deviation (RMSD).

### Ethical approval

This article does not contain any studies with human participants or animals performed by any of the authors.

## Results

### Data mining

**Figure 3:**
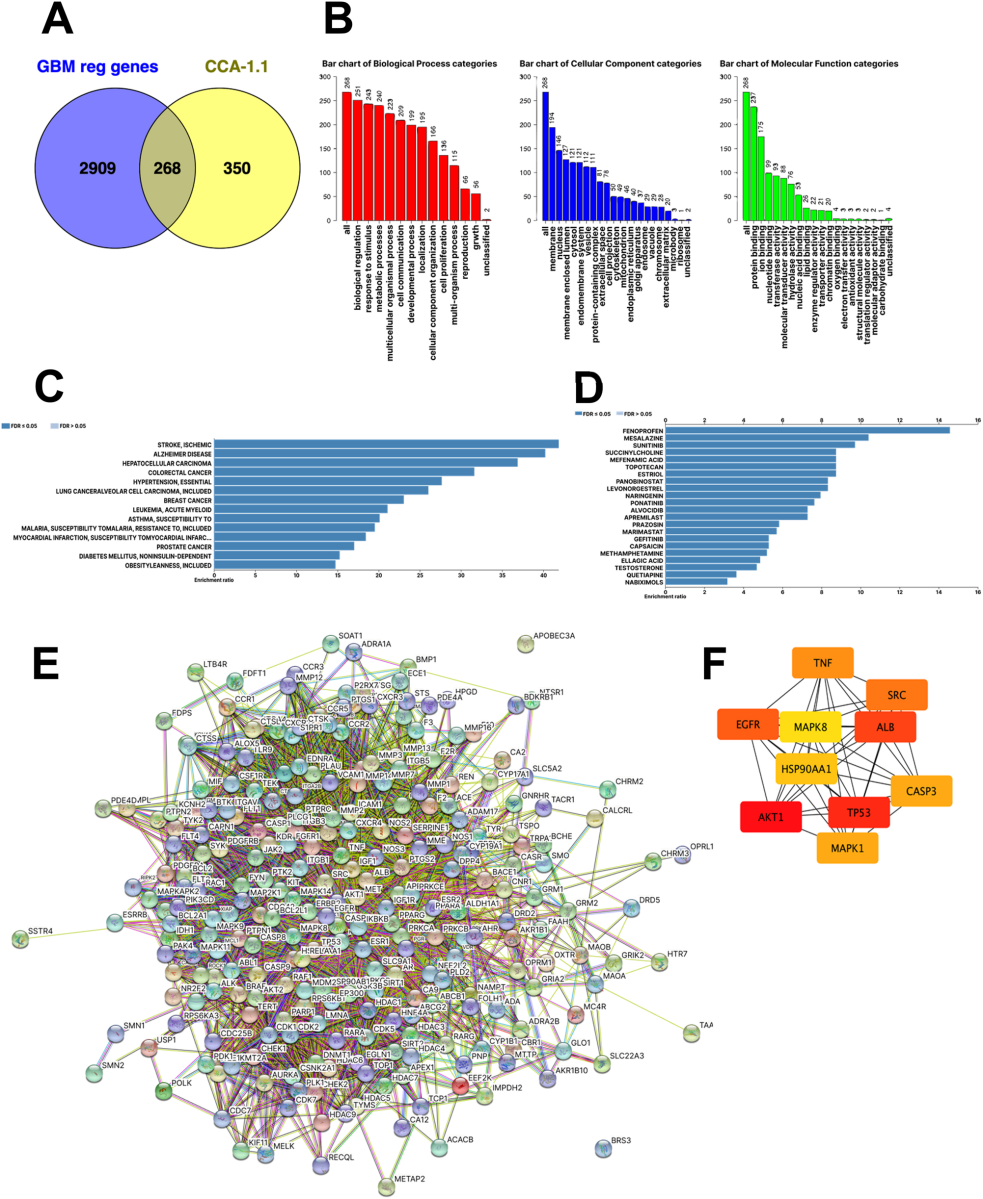
(**A**) Venn diagram showing 268 potential targets of CCA-1.1 against GBM (PTCG). (**B**) Gene Ontology enrichment analysis of the PTCGs. (**C**) Disease–gene association analysis of the PTCGs. (**D**) Drug–gene association analysis of the PTCGs. (**E**) Protein–protein interaction network of the PTCGs. (**F**) Top 10 protein in the network, ranked by degree, as analyzed by CytoScape.

We obtained 100, 4, 9, 494, 134, 71, and 1 target genes of CCA-1.1 using SwissTargetPRediction, SEA Search, MoltarPRed, TargetNet, BindingDB, DINIES, and HitPick, respectively, for a total of 618 predicted targets (Supplementary Table 1). From DISGENET, we collected 3177 GBM-related regulatory genes (Supplementary Table 2). As visualized using a Venn diagram, 268 genes (Fig. 3A, Supplementary Table 3) were targets of CCA-1.1 and involved in the regulation of GBM. These genes were identified as potential targets of CCA-1.1 in glioblastoma (PTCG) and were included in further analyses.

### Functional annotation

A Gene Ontology analysis revealed that PTCGs were involved in various biological processes, including the response to stimulus, metabolic process, and cell communication (Fig. 3B). PTCGs were also enriched for cellular components, including the membrane, nucleus, and cytosol. In addition, PTCGs were associated with terms in the molecular functions category, including protein, ion, and nucleotide binding. A KEGG pathway enrichment analysis revealed that PTCGs were involved in several pathways, such as glioma, pathways in cancer, and p53 signaling pathways (Supplementary Table 4).

### Disease–gene and drug–gene associations

A disease–gene association analysis revealed several diseases related to PTCGs, including Alzheimer’s disease, hepatocellular carcinoma, colorectal cancer, and breast cancer (Fig. 3C). A drug–gene association analysis showed that PTCGs are associated with several drugs, including ABT-869, tyrosine kinase inhibitors (sunitinib, regorafenib, ponatinib, sorafenib, imatinib, and fostamatinib), panobinostat, resveratrol, and tamoxifen (Fig. 3D).

### Protein–protein interaction network and hub gene identification

Using STRING, we constructed a PPI network including 268 nodes and 4597 edges, with an average node degree of 34.3, an average local clustering coefficient of 0.523, and a PPI enrichment *p* value of < 1.0e − 16 (Fig. 3E). Hub genes were selected using the cytoHubba plugin of Cytoscape as the top ten target genes with respect to degree scores, including *AKT1, TP53, ALB, EGFR, SRC, TNF, CASP3, MAPK1, HSP90AA1*, and *MAPK8* (Fig. 3F, Table 1).

**Table 1:** Top 10 proteins in the protein–protein interaction network, ranked by degree, as analyzed by CytoScape.

Rank	Gene symbol	Gene name	Score
1	AKT1	AKT serine/threonine kinase 1	165.0
2	TP53	Tumor protein p53	156.0
3	ALB	Albumin	143.0
4	EGFR	Epidermal growth factor receptor	135.0
5	SRC	SRC proto-oncogene, non-receptor tyrosine kinase	131.0
6	TNF	Tumor necrosis factor	128.0
7	CASP3	Caspase 3	125.0
7	MAPK1	Mitogen-activated protein kinase 1	125.0
9	HSP90AA1	Heat shock protein 90 alpha family class A member 1	112.0
10	MAPK8	Mitogen-activated protein kinase 8	111.0

### Analysis of genetic alterations in hub genes

**Figure 4:**
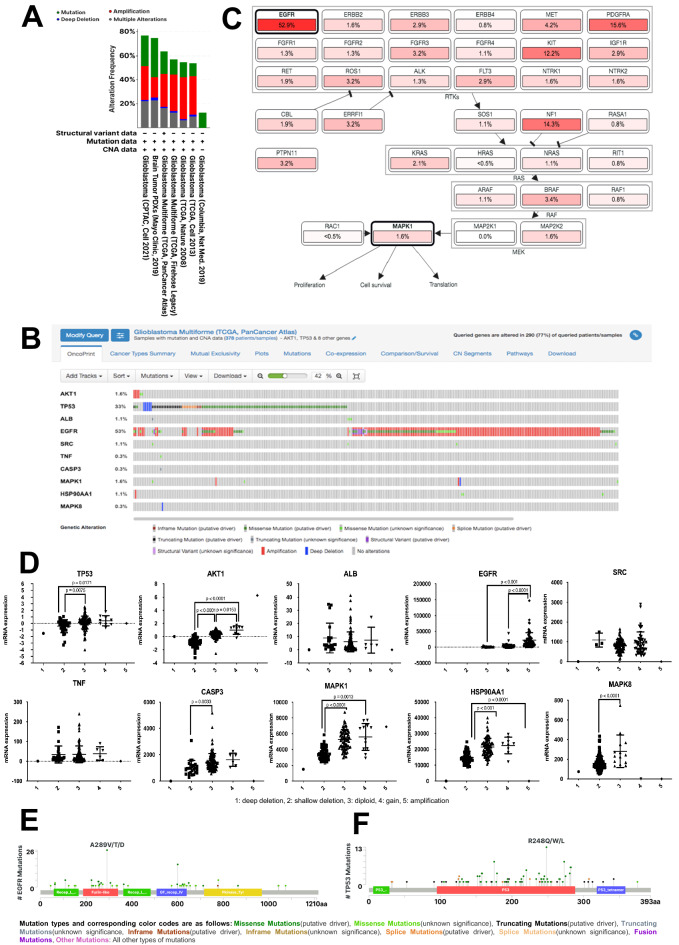
(**A**) Summary of alterations in 10 PTCG reported in GBM studies using cBioportal. (B) OncoPrint analysis of 10 PTCGs in patients with GBM from TCGA PanCancer Atlas study, as analyzed using cBioportal. (**C**) Pathway enrichment analysis related to genetic alterations in 10 PTCGs in patients with GBM from TCGA PanCancer Atlas, as analyzed using cBioportal. (**D**) Copy number alterations of 10 PTCGs in patients with GBM from TCGA PanCancer Atlas, as analyzed using cBioportal. Statistical analysis was performed using one-way ANOVA with Tukey’s multiple comparison test. Mutation profiles of (**E**) EGFR and (**F**) TP53 in patients with GBM from TCGA PanCancer Atlas, as analyzed using cBioportal.

Genetic alterations in the ten hub genes were evaluated based on six studies of GBM using cBioportal (Fig. 4A). TCGA PanCancer Atlas^68^ which showed the second highest genetic alterations and the largest number of patients among the GBM studies and was selected for further analysis. We found mutation rates of 0.3–53% in hub genes in the study population, including *CASP3* (0.3%), *MAPK8* (0.3%), *TNF* (0.3%), *ALB* (1.1%), *SRC* (1.1%), *HSP90AA1* (1.1%), *AKT1* (1.6%), *MAPK1* (1.6%), *TP53* (33%), and *EGFR* (53%) (Fig. 4B). In a mutual exclusivity analysis, three gene pairs were significant, namely *TP53*–*EGFR*, *ALB*–*SRC*, and *TNF*–*CASP3* (Table 2). A pathway enrichment analysis revealed that several pathways are affected by the observed genetic alterations, including RTK-RAS, TP53, PI3K, and cell cycle pathways (Supplementary Table 5). The RTK-RAS pathway was detected in two queries, *EGFR* and *MAPK1*, as well as neighboring genes, including members of the ERBB family, RAS family, and RAF family, which are involved in cellular processes including proliferation, cell survival, and translation (Fig. 4C).

**Table 2:** Mutual exclusivity analysis, as performed using cBioportal.

A	B	*p* value	Tendency
*TP53*	*EGFR*	< 0.001	Mutual exclusivity
*ALB*	*SRC*	< 0.001	Co-occurrence
*TNF*	*CASP3*	0.003	Co-occurrence

Copy number alterations in *ALB, SRC*, and *TNF* were not obvious (Fig. 4D). In *AKT1*, significant differences in mRNA levels were found between alteration types (i.e., shallow deletion, diploid, and gain); in particular, the expression of *AKT1* was highest in cases with copy number gain, followed by diploid, and shallow deletion. The mRNA levels of *TP53* in the shallow deletion group were significantly lower than those in the diploid and gain groups. In *EGFR*, we found that mRNA expression levels in the case of amplification were significantly higher than that those in the diploid and gain groups. mRNA levels of *CASP3* and *MAPK8* in diploids were significantly higher than those in the shallow deletion group. In addition, mRNA levels of *MAPK1* and *HSP90AA1* were significantly higher in the case of gain than in the diploid and shallow deletion groups. We then evaluated *TP53* and *EGFR* mutations across patient samples in Liu et al. (2018). We found several mutations in TP53 in the p53 tetramerization domain, p53-DNA binding domain, and p53 transactivation domain (Fig. 4E). EGFR mutations occurred in many domains, such as the receptor-ligand domain, furin-like cysteine-rich region, growth factor receptor domain IV, and protein tyrosine kinase domain (Fig. 4F).

### Expression of hub genes in glioblastoma samples

**Figure 5:**
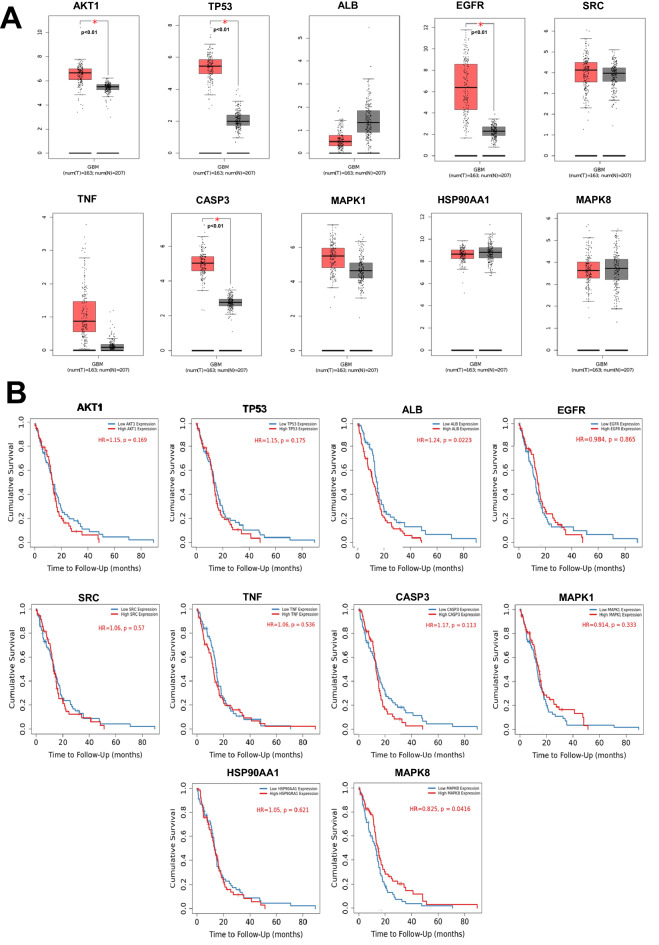
(**A**) Gene expression analysis of 10 PTCGs in patients with GBM and adjacent normal tissues from TCGA, as analyzed using GEPIA. (**B**) Relationships between the overall survival of patients with GBM and the expression of 10 PTCGs, as analyzed using TIMER 2.0.

The mRNA levels of the hub genes *AKT1, TP53, EGFR,* and *CASP3* were significantly higher in patients with GBM than in normal brain tissues (Fig. 5A). In addition, mRNA levels of *ALB, SRC, TNF, MAPK1, HSP90AA1,* and *MAPK8* were not statistically significant between GBM and normal brain tissues.

### Survival analysis of hub genes

The prognostic value of each hub gene was analyzed using a Kaplan–Meier plot. Among the hub genes, only *ALB* and *MAPK8* levels were significantly associated with the survival of patients with GBM (Fig. 5B). Patients with low levels of *ALB* had a better overall survival than that of patients in group with high expression (*p* = 0.0223), whereas patients with high levels of *MAPK8* had a better overall survival than that of patients with low expression levels (*p* = 0.0416).

### Correlation between immune cell infiltration and hub genes

We explored correlations between the expression of hub genes and levels of immune cell infiltration in GBM using the TIMER 2.0 database (Table 3, Supplementary Fig. 1). We selected only four hub genes, *AKT1, TP53, EGFR,* and *CASP3*, based on their high expression levels in GBM, as analyzed by GEPIA. The expression levels of *AKT1* (*r* = 0.311; *p* = 2.06 × 10^−4^), *TP53* (*r* = 0.311; *p* = 7.36 × 10^−5^), *EGFR* (*r* = 0.288; *p* = 6.15 × 10^−4^), and *CASP3* (*r* = 0.232; *p* = 6.24 × 10^−4^) were significantly and positively correlated with purity. Only *CASP3* was significantly negatively correlated with B cells (*r* = −0.181; *p* = 3.44 × 10^−2^). The expression of CD8 + was significantly negatively correlated with the levels of *AKT1* (*r* = −0.318; *p* = 1.53 × 10^−4^) and *EGFR* (*r* = −0.142; *p* = 9.75 × 10^−2^). CD4 + levels were significantly positively correlated with the levels of *AKT1* (*r* = 0.187; *p* = 2.87 × 10^−2^), *TP53* (*r* = 0.192; *p* = 2.49 × 10^−2^), and *EGFR* (*r* = 0.195; *p* = 2.27 × 10^−2^). Macrophage cells were significantly positively correlated with *AKT1* expression (*r* = 0.219; *p* = 1.01 × 10^−2^), *TP53* (*r* = 0.176; *p* = 3.92 × 10^−2^), and *EGFR* (*r* = 0.227; *p* = 7.69 × 10^−3^). Neutrophils were significantly positively correlated with levels of *AKT1* (*r* = 0.266; *p* = 7.85 × 10^−3^) and *TP53* (*r* = 0.248; *p* = 3.42 × 10^−3^). Dendritic cells showed significant positive correlations with levels of *AKT1* (*r* = 0.439; *p* = 8.24 × 10^−8^), *TP53* (*r* = 0.255; *p* = 2.63 × 10^−3^), *EGFR* (*r* = 0.251; *p* = 3.15 × 10^−3^), and *CASP3* (*r* = 0.198; *p* = 2.01 × 10^−2^). Cancer-associated fibroblasts (CAFs) showed significant positive correlations with levels of *AKT1* (*r* = 0.211; *p* = 1.34 × 10^−2^), *TP53* (*r* = 0.211; *p* = 1.35 × 10^−2^), and *CASP3* (*r* = 0.226; *p* = 7.88 × 10^−3^).

**Table 3:** Immune cell infiltration related to the expression levels of *AKT1*, *TP53*, *EGFR*, and *CASP3*.

Gene Name	Parameters	Purity	B cell	CD8 +	CD4 +	Macrophage	Neutrophil	Dendritic cell	Cancer Associated Fibroblast
*AKT1*	R	**0.311**	0.013	**−0.318**	**0.187**	**0.219**	**0.266**	**0.439**	**0.211**
	*P* value	**2.06e-04**	8.78e-01	**1.53e-04**	**2.87e-02**	**1.01e-02**	**7.85e-03**	**8.24e-08**	**1.34e-02**
*TP53*	R	**0.311**	0.135	−0.008	**0.192**	**0.176**	**0.248**	**0.255**	**0.211**
	P value	**7.36e-05**	1.15e-01	9.25e-01	**2.49e-02**	**3.96e-02**	**3.42e-03**	**2.63e-03**	**1.35e-02**
*EGFR*	R	**0.288**	0.07	**−0.142**	**0.195**	**0.227**	**0.053**	**0.251**	0.089
	P value	**6.15e-04**	4.17e-01	**9.75e-02**	**2.27e-02**	**7.69e-03**	5.35e-01	**3.15e-03**	3.00e-01
*CASP3*	R	**0.232**	**−0.181**	0.033	−0.103	−0.019	0.088	**0.198**	**0.226**
	*P* value	**6.24e-03**	**3.44e-02**	7.05e-01	2.31e-01	8.29e-01	3.04e-01	**2.01e-02**	**7.88e-03**

Significant values are in bold.

### CCA-1.1 performed cytotoxicity and induces the modulation of EGFR on U87 glioblastoma cells

**Figure 6:**
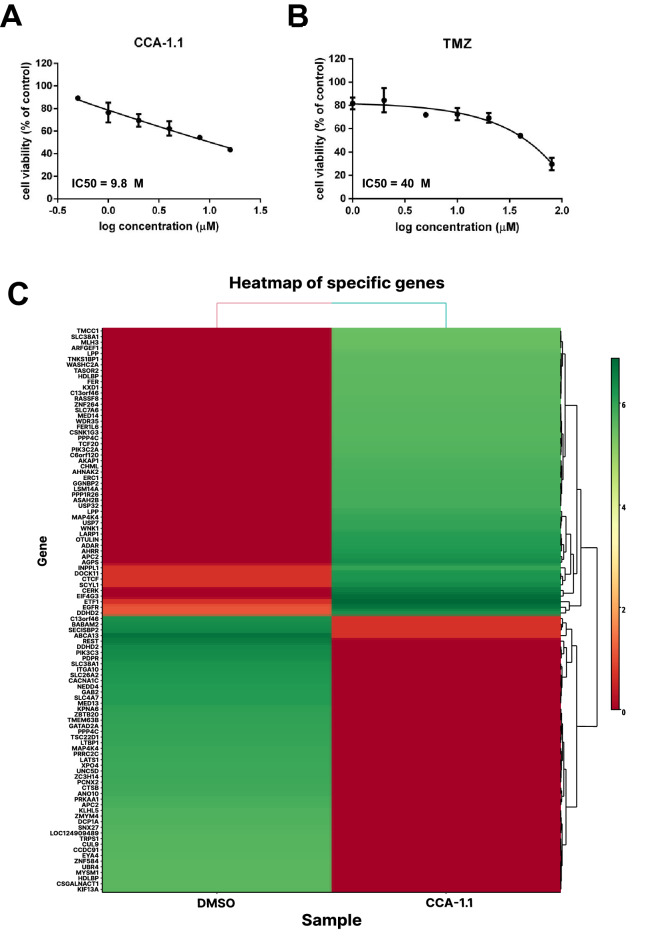
(**A**) Cytotoxicity of CCA-1.1 and (**B**) TMZ in U87 glioblastoma cells. Cytotoxicity was determined using an MTT assay and presented as cell viability as explained in the methods section. Results are shown as the average of the three independent experiments (mean ± SD). (**C**) Heat map of top 100 DEGs between the U87 cells treated with CCA-1.1 and the DMSO.

We performed an MTT assay to measure the cytotoxicity of CCA-1.1 and TMZ, and both compounds showed cytotoxicity against U87 cells with an IC50 value of 9.8 and 40 μM, respectively (Fig. 6A,B). To check the molecular mechanism of CCA-1.1 in U87 cells, we performed next generation sequencing between untreated and CCA-1.1 treated U87 cells, and then analyzed the results for DEGs (Fig. 6C, Supplementary Table 6). The raw data of gene expression can be accessed at the Gene Expression Omnibus (GEO, http://www.ncbi.nlm.nih.gov/geo/), using accession number GSE206241. Among the potential target genes, only EGFR showed significant results based on differential expression analysis, in which EGFR transcript variant 8 was upregulated in CCA-1.1 treated U87 cells, whereas EGFRvIII was downregulated in U87 cells after treatment with CCA-1.1 (Table 4). These findings confirm the bioinformatic approach which highlights the importance of EGFR as targets of CCA-1.1 in inhibition of GBM.

**Table 4:** Differentially expressed genes of hub genes in U87 glioblastoma cells upon treatment of CCA-1.1.

No	Gene symbol	Ref seq	Gene name	Log FC	*p* value
1	EGFR	NM_001346900.2	Epidermal growth factor receptor (EGFR), transcript variant 8, mRNA	**8.83688311**	**1.03E-05**
		NM_001346941.2	Epidermal growth factor receptor (EGFR), transcript variant EGFRvIII, mRNA	**−6.111141843**	**0.00063995**
		NM_001346898.2	Epidermal growth factor receptor (EGFR), transcript variant 6, mRNA	−2.651710224	0.10586144
		NR_047551.1	EGFR antisense RNA 1 (EGFR-AS1), long non-coding RNA	2.325569699	0.30560263
		NM_201283.2	Epidermal growth factor receptor (EGFR), transcript variant 3, mRNA	−0.969886184	0.5118575
		NM_001346899.2	Epidermal growth factor receptor (EGFR), transcript variant 7, mRNA	−0.816340926	0.56093641
		XM_047419953.1	Epidermal growth factor receptor (EGFR), transcript variant X2, mRNA	−0.777241106	0.59354503
		NM_001346897.2	Epidermal growth factor receptor (EGFR), transcript variant 5, mRNA	0.155644698	1
2	TP53	not found	Not found	Not found	Not found
3	AKT1	NM_001014431.2	AKT serine/threonine kinase 1 (AKT1), transcript variant 3, mRNA	−0.173979481	0.8970585
4	ALB	not found	Not found	Not found	Not found
5	SRC	not found	Not found	Not found	Not found
6	TNF	NM_016292.3	TNF receptor associated protein 1 (TRAP1), transcript variant 1, mRNA; nuclear gene for mitochondrial product	0.095069258	0.94426958
7	CASP3	not found	Not found	Not found	Not found
8	MAPK1	NM_138957.3	Mitogen-activated protein kinase 1 (MAPK1), transcript variant 2, mRNA	0.095103156	0.94434009
9	HSP90AA1	NM_005348.4	Heat shock protein 90 alpha family class A member 1 (HSP90AA1), transcript variant 2, mRNA	−0.088807883	0.94722009
10	MAPK8	NM_001323330.2	Mitogen-activated protein kinase 8 (MAPK8), transcript variant 16, mRNA	−0.03700038	1

Significant values are in bold.

### Prediction of effects of mutations on protein function

We identified EGFR as a promising target of CCA-1.1 for GBM treatment. We further predicted the functional effects of *EGFR* alterations using several databases, including PolyPhen-2, Provean, SIFT, FATHMM, and PANTHER. We selected 22 EGFR mutations detected in GBM samples by Liu et al. (2018) (TCGA PanCancer); these mutations were located in the growth factor receptor domain, protein kinase-like (PK-like), receptor L domain, growth factor receptor domain IV, furin-like cysteine-rich region, protein kinase-like (PK-like), and protein tyrosine kinase (Table 5, Supplementary Table 7). The *EGFR* mutations in the protein kinase-like domain, namely E709K, V774M, and L861Q, were predicted to be damaging, deleterious, and cancer-related (Table 5). Another mutation, T263P, located in a furin-like cysteine-rich region, was also predicted to be associated with cancer. The V774M mutation, which occurs in the protein kinase-like domain, was predicted to be damaging and associated with cancer. In addition, L861Q, in the protein tyrosine kinase domain, was predicted to be damaging and related to cancer.

**Table 5:** Prediction of EGFR mutations and activity.

No	Mutant	PolyPhen-2 HumDiv		PolyPhen HumVar		Provean		SIFT		FATHMM					PANTHER		
		Score	Prediction	Score	Prediction	Score	Prediction	Score	Prediction	Domain Involved	Cancer		Inherited disease				
											Score	Prediction	Score	Prediction	Preservation time	Message	Pdel
1	A289D	0.996	PD	0.993	PD	−5.24	Del	0.001	D	Growth factor receptor domain	−1.01	Cancer	−0.01	T	797	PD	0.74
2	A289T	0.987	PD	0.974	PD	−3.54	Del	0.001	D	Growth factor receptor domain	−1.02	Cancer	−0.02	T	797	PD	0.74
3	A289V	0.997	PD	0.989	PD	−3.56	Del	0.001	D	Growth factor receptor domain	−1.04	Cancer	−0.04	T	797	PD	0.74
4	C620W	1	PD	0.998	PD	−10.32	Del	0	D	Growth factor receptor domain	−3.32	Cancer	0.00	T	911	PD	0.85
5	C620Y	1	PD	0.989	PD	−10.32	Del	0	D	Growth factor receptor domain	−3.3	Cancer	0.02	T	911	PD	0.85
6	E709K	0.974	PD	0.721	Pos D	−3.38	Del	0.003	D	Protein kinase−like (PK−like)	−1.93	Cancer	0.07	T	842	PD	0.78
7	G598V	0.997	PD	0.849	Pos D	−8.43	Del	0.004	D	Growth factor receptor domain	−2.26	Cancer	1.06	T	797	PD	0.74
8	H304Y	0	benign	0.005	benign	−2.01	Neut	1	T	Growth factor receptor domain	−1.02	Cancer	−0.02	T	456	PD	0.57
9	L62R	0.795	Pos D	0.553	Pos D	−2.02	Neut	0.006	D	Receptor L domain	−0.6	Passenger/Other	−1.27	T	455	PD	0.57
10	P596R	1	PD	0.999	PD	−8.44	Del	0	D	Growth factor receptor domain IV	−3.96	Cancer	−0.38	T	911	PD	0.85
11	P596S	1	PD	0.968	PD	−7.5	Del	0.005	D	Growth factor receptor domain IV	−3.93	Cancer	−0.35	T	911	PD	0.85
12	R108G	1	PD	1	PD	−5.87	Del	0.01	D	Receptor L domain	−0.81	Cancer	−1.48	T	842	PD	0.78
13	R108K	1	PD	1	PD	−2.59	Del	0.001	D	Receptor L domain	−0.78	Cancer	−1.44	T	842	PD	0.78
14	R222C	1	PD	1	PD	−6.52	Del	0	D	Growth factor receptor domain	−1.05	Cancer	−0.05	T	797	PD	0.74
15	R252C	1	PD	0.993	PD	−3.25	Del	0.025	D	Furin−like cysteine rich region	−2.74	Cancer	−2.08	D	456	PD	0.57
16	R252P	0.998	PD	0.991	PD	−3.17	Del	0.058	T	Furin−like cysteine rich region	−2.73	Cancer	−2.06	D	456	PD	0.57
17	S645C	0.999	PD	0.982	PD	−1.96	Neut	0.187	T	−	−1.07	Cancer	−0.94	T	361	Pos D	0.5
18	T263P	0.952	Pos D	0.913	PD	−1.38	Neut	0.087	T	Furin−like cysteine rich region	−2.51	Cancer	−1.85	D	176	Prob Benign	0.27
19	T363I	1	PD	0.994	PD	−5.07	Del	0.001	D	Receptor L domain	−0.96	Cancer	−1.62	D	842	PD	0.78
20	V774M	1	PD	0.994	PD	−1.61	Neutral	0.001	D	Protein kinase−like (PK−like)	−2.3	Cancer	−0.3	T	456	PD	0.57
21	L861Q	1	PD	0.993	PD	−5.29	Del	0.008	D	Protein tyrosine kinase	−1.88	Cancer	−1.59	D	797	PD	0.74
22	H773_V774dup	NA	NA	NA	NA	−10.34	Del	NA	NA	NA	NA	NA	NA	NA	NA	NA	NA

*PD* probably damaging, *Pos D* possibly damaging, *Del* Deleterious, *Neut* Neutral, *D* Damaging, *T* Tolerated.

### Molecular docking and MD

**Figure 7:**
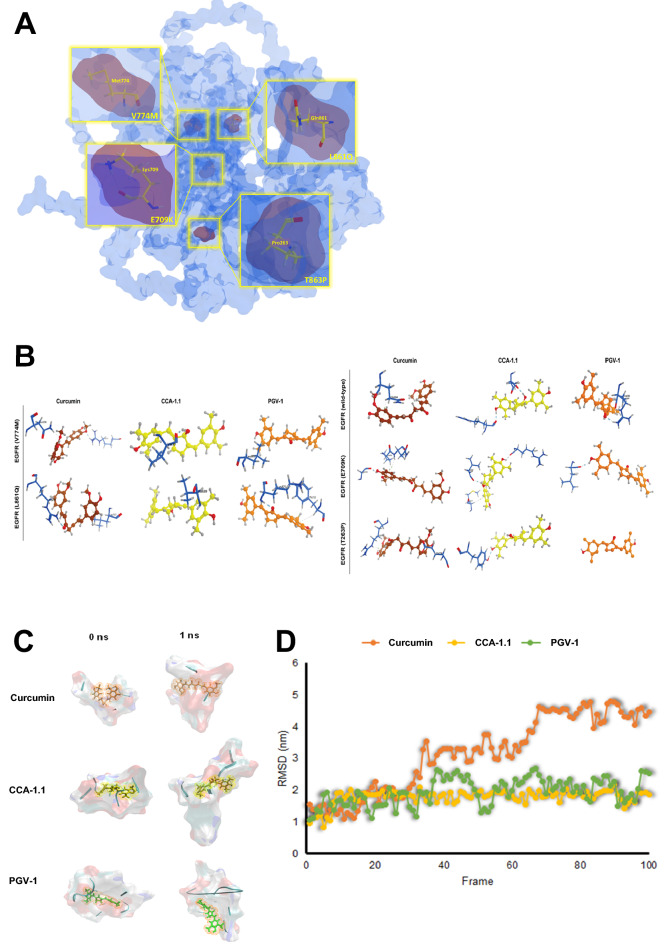
(**A**) 3D visualization of EGFR mutations, E709K (Glu709 Lys709), T263P (Thr263 Pro263), V774M (Val774 Met774), and L861Q (Leu861 Gln861). (**B**) Visualization of molecular docking results for wild-type EGFR and mutant EGFR (E709K, T263P, V774M, and L861Q) against Curcumin, CCA-1.1, and PGV-1. (**C**) Visualization of the binding interaction of compounds (Curcumin, CCA-1.1, and PGV-1) against mutant EGFR L861Q at the initial time and after 1 ns MD simulation. (**D**) Root mean squared deviation (RMSD) of compounds (Curcumin, CCA-1.1, and PGV-1) in complex with mutant EGFR L861Q after 1 ns MD, shown in 100 frames.

We successfully predicted the structures of mutant EGFR using a template from AlphaFold (Supplementary Fig. 2). Four mutants were selected from previous experiments. Each complex protein (Fig. 7A) was docked against curcumin and its analogues, PGV-1 and CCA-1.1. The molecular docking results showed that in wild-type EGFR, PGV-1 had the lowest docking score of -13.87 kcal/mol and formed one hydrogen bond with Arg686 (Fig. 7B, Table 6). For the E709K and V774M mutant forms of EGFR, curcumin had the lowest binding energy of −11.74 kcal/Mol with three hydrogen bonds (Gly696, Pro699, and Asn700) and −11.94 kcal/Mol with two hydrogen bonds (Asn298 and Arg831), respectively. CCA-1.1 showed the lowest docking scores of −11.29 and −12.62 kcal/Mol in the T263P and L861Q mutant forms of EGFR, respectively. Interestingly, for all mutant forms, CCA-1.1 showed better binding affinity than PGV-1 (Table 6). CCA-1.1 also had much stronger binding activity (ΔG = -12.62 kcal/Mol) for the L861Q mutant than wild-type EGFR, while PGV-1 did no show a difference between mutant and wild-type EGFR. These results show that CCA-1.1 performs better than PGV-1 in the inhibition of mutant EGFR (E709K, T263P, V774, and L861Q). Taken together, these results indicate that CCA-1.1 can inhibit many EGFR variants.

**Table 6:** Molecular docking scores for curcumin, CCA-1.1, and PGV-1 against wild-type and mutant EGFR.

Drug	Protein	Binding Energy (kcal/Mol)	No of H bonds	Interacting amino acids (Distance)
Curcumin	EGFR (wild-type)	−11.05	1	Gln684 (2.97)
	EGFR (E709K)	−11.74	3	Gly696 (2.21), Pro699 (3.08), Asn700 (3.44)
	EGFR (T263P)	−10.12	2	Arg686 (2.98), Glu736 (3.19)
	EGFR (V774M)	−11.94	2	Asn298 (1.84), Arg831 (2.13)
	EGFR (L861Q)	−10.51	2	Arg297 (1.92), Leu707 (3.00)
CCA-1.1	EGFR (wild-type)	−12.51	2	Arg53 (1.80), Glu687 (1.79)
	EGFR (E709K)	−11.02	3	Val30 (2.04), Met54 (3.76), Arg297 (1.93)
	EGFR (T263P)	−11.29	1	Tyr299 (2.02)
	EGFR (V774M)	−10.91	1	Val30 (2.09)
	EGFR (L861Q)	−12.62	1	Val689 (3.13)
PGV-1	EGFR (wild-type)	−13.87	1	Arg686 (1.84)
	EGFR (E709K)	−10.73	1	Leu777 (2.29)
	EGFR (T263P)	−10.37	–	–
	EGFR (V774M)	−10.58	1	Leu861 (3.09)
	EGFR (L861Q)	−11.82	2	Phe723 (2.12), Lys875 (2.85)

The results of molecular docking were validated using MD simulation. As the representative, we chose the binding pocket of EGFR L861Q in complex with curcumin, PGV-1, and CCA-1.1. After a 1-ns simulation, CCA-1.1 displayed a minor change in the position and binding trajectory with mutant EGFR L861Q, which indicates the most stable interaction (Fig. 7C). In the presence of PGV-1, the binding pocket of mutant EGFR L861Q showed more change in position than in CCA-1.1. Further, a more dynamic change was observed with curcumin, which clarified the less stable interaction of curcumin and PGV-1 than that of CCA-1.1 (Fig. 7C). Higher-binding stability of CCA-1.1 compared with that of PGV-1 and curcumin was also demonstrated by the RMSD value of each compound after the 1-ns MD simulation. CCA-1.1 demonstrated a stable RMSD value around 1.8 nm (Fig. 7D). An increase in the RMSD value up to 2.4 and 4.6 nm was shown by PGV-1 and CCA-1.1, respectively, which demonstrates a less stable binding interaction (Fig. 7D). The results of the MD simulation confirmed the validity of the molecular docking study, indicating CCA-1.1 as the most effective EGFR inhibitor.

## Discussion

We identified four targets of CCA-1.1 in GBM (i.e., TP53, EGFR, AKT1, and CASP3) by an integrative bioinformatics analysis. *TP53* encodes the P53 protein, a tumor suppressor that inhibits cancer cell proliferation and promotes apoptosis^69^. *TP53* is frequently mutated in GBM, and these mutations are mainly deletions, affecting P53 function and thereby triggering cancer progression. We also detected copy number gains, suggesting an increase in p53 expression. Both curcumin and PGV-1 compounds have been shown to increase p53 expression in breast cancer cells^70^. Further studies of changes in p53 expression in response to CCA-1.1 treatment in GBM are needed to support the findings of this study.

Mutations in p53 are found in almost half of human cancers^71^, a loss of p53 function promotes invasion, metastasis, and chemoresistance^72^. Mutations in p53, particularly gain-of-function mutations, increase the inflammatory response in patients with GBM^73^. AKT1 is a protein serine/threonine kinase that plays a role in the PI3K/AKT pathway, which regulates cell proliferation and survival^74^. The dysregulation of AKT is common in cancer, with reports of epigenetic modifications, mutations, and overexpression^75,76^. PI3K/Akt is a highly targeted pathway for glioblastoma therapy^77^. Several previous studies have explored the AKT-targeted anticancer effects of curcumin and its analogs. Curcumin may be effective in combination with TMZ in GBM^78^. Yin reported that curcumin increases the effectiveness of temozolomide against U87 glioblastoma cells by increasing ROS levels, inhibiting AKT/mTOR signaling, and promoting apoptosis^79^. Curcumin inhibits GBM via the pRb, p53, JAK/STST, MAPK, PI3K/Akt, and NF-κB pathways^80^. Another analog of curcumin, C-150, inhibits GBM progression by targeting the NF-κB, Notch, and Akt pathways^81^. Previous research on curcumin and PGV-1 has shown that these compounds inhibit PI3K/AKT signaling in breast cancer cells and colon cancer cells. PGV-1 inhibits NF-κB activation^82^ which is related to the PI3K/Akt pathway. Elucidating the mechanism by which CCA-1.1 influences the PI3K/AKT pathway will provide a scientific basis for its utilization as an anti-GBM agent.

*CASP3* encodes caspase 3, which contributes to the final steps in apoptosis, and is also called an executioner caspase^83^. Increased caspase-3 expression in triggers GBM cell death^84^. The inhibition of caspase-3 in brain-resident immune cells promotes GBM progression^85^. Previous studies have shown that both curcumin and PGV-1 trigger apoptosis by increasing caspase expression. The curcumin analogs PGV-0 and PGV-1 stimulate the apoptosis of T47D breast cancer cells by the activation of Caspase-3^86^. Further studies of the effect of CCA-1.1 on caspase 3 expression and activity are needed.

*EGFR* encodes the human epidermal growth factor receptor, a member of the tyrosine kinase receptor family^87^. Mutations in EGFR activate EGFR signaling, which triggers proliferation and survival in GBM^88^. EGFR mutations have been found in 53% of patients with GBM^68^, including gains or amplifications, suggesting an increase in EGFR expression. Several compounds successfully inhibit EGFR signaling, for example, Higenamine^89^, 20(R, S)-protopanaxatriol, a metabolite from protopanaxatriol ginsenosides^90^, and Tubeimoside-I, which increases the sensitivity of glioblastoma cells towards temozolomide^91^.

Extensive research has focused on the effects of curcumin and its analogs targeting EGFR in cancer cells. Curcumin inhibits EGFR signaling and reduces EGFR expression in cancer cells. Curcumin increases sensitivity to gefitinib by inhibiting EGFR signaling in non-small cell lung cancer^92^. In addition, curcumin enhances the anticancer activity of gefitinib in vitro and in vivo in lung cancer by inducing EGFR degradation^93^. Curcumin downregulates EGFR in colon cancer cells by reducing the transcription factor EGR1^94^. Another study has shown that curcumin inhibits the autophosphorylation of EGFR^95^. Starok et al. showed that curcumin has dual effects on EGFR by inhibiting enzymatic activity of the EGFR tyrosine kinase domain and by entering the lipid bilayer, thus affecting EGFR dimerization^96^. A recent study by Ali et al. has shown that curcumin analog 3c has a greater inhibitory effect on leukemic cells than those of curcumin and gefitinib, and this analog inhibits EGFR activity^97^.

Mutations in the EGFR kinase domain have been shown to cause constitutively active ligand-independent signaling^98^ and to affect the sensitivity of glioma cells to temozolomide^99^. E709K is a mutation in EGFR exon 18 responsible for lung cancer cell resistance to gefitinib, erlotinib, AZD9291, and CO1686^100^. It is a rare type of EGFR mutation in lung cancer^101^. The T263P mutation is located in the extracellular domain of EGFR, which leads to ligand-independent signaling activation^102^ and tumor progression in GBM^103^. Moreover, the T263P EGFR mutant form has a furin-like cysteine-rich (FU-CR) domain involved in signal transduction, including an important role in promoting Wnt/β-catenin signaling^104–107^. L861Q is a missense mutation in the EGFR kinase domain of GBM^108^. The L861Q mutation increases kinase activity and tumor progression but does not increase the sensitivity of tumor cells to EGFR tyrosine kinase inhibitors^109^. The EGFR V774M mutation is associated with non-small-cell lung cancer progression^110^ and resistance to tyrosine kinase inhibitors^111^. A missense mutation in the EGFR kinase domain, V774M, which leads to amplification, has also been found in Japanese patients with GBM^112^. V774M is considered a functional mutation in lung cancer^113^.

In a molecular docking analysis, CCA-1.1 showed a lower docking score than that of PGV-1 in wild-type and mutant EGFR E709K, T263P, and L861Q and slightly higher docking scores for V774M. The molecular docking results for wild-type EGFR are supported by previous studies. PGV-1 exhibits the weakest interaction with EGFR and HER2 in silico^114^. Interestingly, CCA-1.1 showed a similar or better interaction with EGFR than PGV-1^28^. Further, MD simulation demonstrated a more stable binding interaction of CCA-1.1 during the 1-ns simulation compared to the binding of PGV-1 and curcumin. Thus, clarifying the results of the molecular docking study. Therefore, further research on CCA-1.1 targeting EGFR is very important for its development as an anti-GBM agent.

GBM gene profiling has revealed three GBM subtypes: proneural (TCGA-PN), classical (TCGA-CL), and mesenchymal (TCGA-MES)^115^. GBM subtypes are characterized by abnormalities in platelet-derived growth factor alpha (PDGFRA), isocitrate dehydrogenase1 (IDH1), epidermal growth factor receptor (EGFR), and neurofibromin1 (NF1)^116^. Different subtypes may respond differently to therapies and show differences in the immune microenvironment^117^. Several studies have suggested that mesenchymal GBM is the most immunogenic, proinflammatory subtype, characterized by significant M2 macrophage and neutrophil gene expression^118,119^. Therefore, we expected to observe correlations between the expression of the four hub genes and the level of immune cell infiltration in GBM. In general, immune cell infiltration can be classified into two types: (1) activation of the immune response by pro-inflammatory cells and CD8 + cytotoxic T lymphocytes (CTL) and (2) suppression of the immune response to cancer cells, e.g., by regulatory T cells (Tregs). Considering the complexity of GBM and the presence of the blood–brain barrier, it is plausible that the immune response is strictly regulated, resulting in extensive immune cell infiltration^120–122^. Both adaptive and innate tumor-infiltrating immune cells are involved, i.e., B cells, CD8 + , and CD4 + cells as well as macrophages, neutrophils, and dendritic cells (DCs), respectively^123^. AKT1 and EGFR negatively affected CD8 + , while B cells were negatively correlated with CASP3 expression levels (with correlation coefficients of < 0.5). Positive correlations were observed between the expression levels of AKT1, TP53, and EGFR and the frequencies of CD4 + cells and all of the above-mentioned innate immune cells. CASP3 expression was positively correlated with DCs. Despite the low frequency of fibroblasts in the healthy brain, CAFs are found in GBM^124,125^. Here, we found that CAFs are positively related to AKT1, TP53, and CASP3 expression. Mu et al. reported that CD4 + plays a role in angiogenesis and the progression of GBM^126^. We propose that targeting the four newly identified gene candidates may be an effective approach to alter the immune response to cancer.

The cytotoxicity assay of CCA-1.1 and TMZ showed that CCA-1.1 has a better cytotoxicity than TMZ based on the IC50 values, in which the cytotoxicity against U87 cells with an IC50 value are 9.8 uM for CCA-1.1 and 40 uM for TMZ, indicating high potency of CCA-1.1 for GBM therapy. DEGs showed that among the potential target genes, only EGFR showed significant results, in which the EGFR transcript variant 8 was upregulated in CCA-1.1 treated U87 cells, whereas EGFRvIII was downregulated in U87 cells after treatment with CCA-1.1., indicating the important role of EGFR in the cytotoxicity of CCA-1.1. A previous study showed the heterogeneity of EGFR in glioblastoma cells, also referred to as EGFR truncation variants^127^. Moreover, genetic amplification and mutations in EGFR are the most common oncogenic events in GBM^128^. EGFR is encoded by the EGFR gene, producing mRNA transcript EGFR variant 1, which produces isoform a. In addition to isoform a, EGFR produces several alternatively spliced transcript variants^129^. Several mRNA variants encode EGFR isoforms, such as variants 1 and 8. EGFR transcript variant 1 encodes the full-length protein of EGFR, while variant 8 encodes a shorter protein. A previous study stated that all isoforms encoded by all EGFR variants could interact with their ligand, namely epidermal growth factor (EGF)^130^. Furthermore, Weinholdt explained that only the EGFR1 isoform had been widely studied for its biological function^131^. EGFRvII is an oncogenic EGFR that is responsible for sensitivity to tyrosine kinase inhibitors^127^.

EGFRvIII is an interesting therapeutic target in GBM therapy because EGFRvIII is present in 25–30% of the glioblastoma cell population^132^. EGFRvIII undergoes a 6–273 amino acid deletion at exon 2–7, encoding the extracellular domain of EGFR^133^, and EGFRvIII can undergo dimerization via a ligand-independent activation pathway^132^. EGFRvIII differs from mutant EGFR on the extracellular domain, namely due to the deletion of certain amino acids causing slow receptor internalization, as well as a slower constitutively phosphorylation level compared to EGFR isoform 1^134^. The results of NGS showed the downregulation of EGFRvIII due to CCA-1.1 treatment. This indicates the potential of CCA-1.1 to constitutively inhibit mutant EGFR and its potential as an inhibitor of EGFR in GBM.

According to the NCBI gene bank, EGFR transcript variant 1 has a length of 9950 bp, while EGFRvIII and EGFR transcript variant 8 have a length of 9104 and 9676 bp, respectively. Transcript variant 8 produces an EGFR isoform that is shorter than transcript variant 1 and a distinct N-terminus compared to isoform a. Research on the biological functions of other EGFR transcript variants, including transcript variant 8, are limited. In this present study, the results of NGS showed an increase in mRNA expression of EGFR variant 8 due to CCA-1.1 treatment; however, the biological role of EGFR variant 8 and the mechanism of CCA-1.1 in regulating the expression of this variant requires further study.

This study had several limitations. First, the protein targets of CCA-1.1 were curated or predicted using public databases based on a particular algorithm. Second, the results of the bioinformatics analyses need to be validated by in vitro and in vivo assays as well as clinical trials. Nevertheless, the results of this study are expected to accelerate the development of drugs for GBM.

## Conclusion

Using an integrative bioinformatics approach, four CCA-1.1 targets in GBM were obtained: TP53, EGFR, AKT1, and CASP3. In addition to the potential therapeutic effects of CCA-1.1 mediated by these four proteins and the inhibition of signaling pathways, it also has the potential to modulate the immune environment. A cytotoxicity assay showed that CCA-1.1 has a better cytotoxicity than TMZ with an IC50 value of 9.8 μM compared 40 μM for TMZ. DEGs showed that among the potential target genes, only EGFR showed significant results, in which the EGFR transcript variant 8 was upregulated, whereas EGFRvIII was downregulated in U87 cells after treatment with CCA-1.1. Molecular docking results revealed that CCA-1.1 can inhibit many EGFR mutants in GBM. Further, MD simulation revealed that the binding of CCA-1.1 with the mutant EGFR L861Q is the most stable compared to those of curcumin and PGV-1. These findings require further confirmation with laboratory experiments and clinical trials for the development of GBM therapies.

## Supplementary Information

Supplementary Information 1.

Supplementary Information 2.

Supplementary Information 3.

## Data Availability

All data produced by this study are disclosed in the manuscript and additional files. The raw data of gene expression can be accessed at the Gene Expression Omnibus (GEO, http://www.ncbi.nlm.nih.gov/geo/), using accession number GSE206241.
