# Supplementary material for: Identification of potential targets of the curcumin analog CCA-1.1 for glioblastoma treatment : integrated computational analysis and in vitro study
Source: Sci Rep. 2022 Aug 17;12:13928. doi: 10.1038/s41598-022-18348-9 (PMC9385707; doi:10.1038/s41598-022-18348-9)
Supplement: Supplementary file 1 — Supplementary Information 1. [file 41598_2022_18348_MOESM1_ESM.docx]

**Supplementary Files**

**Amino Acid Sequences of EGFR variants**

**E709K**

MRPSGTAGAALLALLAALCPASRALEEKKVCQGTSNKLTQLGTFEDHFLSLQRMFNNCEVVLGNLEITYVQRNYDLSFLKTIQEVAGYVLIALNTVERIPLENLQIIRGNMYYENSYALAVLSNYDANKTGLKELPMRNLQEILHGAVRFSNNPALCNVESIQWRDIVSSDFLSNMSMDFQNHLGSCQKCDPSCPNGSCWGAGEENCQKLTKIICAQQCSGRCRGKSPSDCCHNQCAAGCTGPRESDCLVCRKFRDEATCKDTCPPLMLYNPTTYQMDVNPEGKYSFGATCVKKCPRNYVVTDHGSCVRACGADSYEMEEDGVRKCKKCEGPCRKVCNGIGIGEFKDSLSINATNIKHFKNCTSISGDLHILPVAFRGDSFTHTPPLDPQELDILKTVKEITGFLLIQAWPENRTDLHAFENLEIIRGRTKQHGQFSLAVVSLNITSLGLRSLKEISDGDVIISGNKNLCYANTINWKKLFGTSGQKTKIISNRGENSCKATGQVCHALCSPEGCWGPEPRDCVSCRNVSRGRECVDKCNLLEGEPREFVENSECIQCHPECLPQAMNITCTGRGPDNCIQCAHYIDGPHCVKTCPAGVMGENNTLVWKYADAGHVCHLCHPNCTYGCTGPGLEGCPTNGPKIPSIATGMVGALLLLLVVALGIGLFMRRRHIVRKRTLRRLLQERELVEPLTPSGEAPNQALLRILK**K**TEFKKIKVLGSGAFGTVYKGLWIPEGEKVKIPVAIKELREATSPKANKEILDEAYVMASVDNPHVCRLLGICLTSTVQLITQLMPFGCLLDYVREHKDNIGSQYLLNWCVQIAKGMNYLEDRRLVHRDLAARNVLVKTPQHVKITDFGLAKLLGAEEKEYHAEGGKVPIKWMALESILHRIYTHQSDVWSYGVTVWELMTFGSKPYDGIPASEISSILEKGERLPQPPICTIDVYMIMVKCWMIDADSRPKFRELIIEFSKMARDPQRYLVIQGDERMHLPSPTDSNFYRALMDEEDMDDVVDADEYLIPQQGFFSSPSTSRTPLLSSLSATSNNSTVACIDRNGLQSCPIKEDSFLQRYSSDPTGALTEDSIDDTFLPVPEYINQSVPKRPAGSVQNPVYHNQPLNPAPSRDPHYQDPHSTAVGNPEYLNTVQPTCVNSTFDSPAHWAQKGSHQISLDNPDYQQDFFPKEAKPNGIFKGSTAENAEYLRVAPQSSEFIGA

**T263P**

MRPSGTAGAALLALLAALCPASRALEEKKVCQGTSNKLTQLGTFEDHFLSLQRMFNNCEVVLGNLEITYVQRNYDLSFLKTIQEVAGYVLIALNTVERIPLENLQIIRGNMYYENSYALAVLSNYDANKTGLKELPMRNLQEILHGAVRFSNNPALCNVESIQWRDIVSSDFLSNMSMDFQNHLGSCQKCDPSCPNGSCWGAGEENCQKLTKIICAQQCSGRCRGKSPSDCCHNQCAAGCTGPRESDCLVCRKFRDEATCKD**P**CPPLMLYNPTTYQMDVNPEGKYSFGATCVKKCPRNYVVTDHGSCVRACGADSYEMEEDGVRKCKKCEGPCRKVCNGIGIGEFKDSLSINATNIKHFKNCTSISGDLHILPVAFRGDSFTHTPPLDPQELDILKTVKEITGFLLIQAWPENRTDLHAFENLEIIRGRTKQHGQFSLAVVSLNITSLGLRSLKEISDGDVIISGNKNLCYANTINWKKLFGTSGQKTKIISNRGENSCKATGQVCHALCSPEGCWGPEPRDCVSCRNVSRGRECVDKCNLLEGEPREFVENSECIQCHPECLPQAMNITCTGRGPDNCIQCAHYIDGPHCVKTCPAGVMGENNTLVWKYADAGHVCHLCHPNCTYGCTGPGLEGCPTNGPKIPSIATGMVGALLLLLVVALGIGLFMRRRHIVRKRTLRRLLQERELVEPLTPSGEAPNQALLRILKETEFKKIKVLGSGAFGTVYKGLWIPEGEKVKIPVAIKELREATSPKANKEILDEAYVMASVDNPHVCRLLGICLTSTVQLITQLMPFGCLLDYVREHKDNIGSQYLLNWCVQIAKGMNYLEDRRLVHRDLAARNVLVKTPQHVKITDFGLAKLLGAEEKEYHAEGGKVPIKWMALESILHRIYTHQSDVWSYGVTVWELMTFGSKPYDGIPASEISSILEKGERLPQPPICTIDVYMIMVKCWMIDADSRPKFRELIIEFSKMARDPQRYLVIQGDERMHLPSPTDSNFYRALMDEEDMDDVVDADEYLIPQQGFFSSPSTSRTPLLSSLSATSNNSTVACIDRNGLQSCPIKEDSFLQRYSSDPTGALTEDSIDDTFLPVPEYINQSVPKRPAGSVQNPVYHNQPLNPAPSRDPHYQDPHSTAVGNPEYLNTVQPTCVNSTFDSPAHWAQKGSHQISLDNPDYQQDFFPKEAKPNGIFKGSTAENAEYLRVAPQSSEFIGA

**V774M**

MRPSGTAGAALLALLAALCPASRALEEKKVCQGTSNKLTQLGTFEDHFLSLQRMFNNCEVVLGNLEITYVQRNYDLSFLKTIQEVAGYVLIALNTVERIPLENLQIIRGNMYYENSYALAVLSNYDANKTGLKELPMRNLQEILHGAVRFSNNPALCNVESIQWRDIVSSDFLSNMSMDFQNHLGSCQKCDPSCPNGSCWGAGEENCQKLTKIICAQQCSGRCRGKSPSDCCHNQCAAGCTGPRESDCLVCRKFRDEATCKDTCPPLMLYNPTTYQMDVNPEGKYSFGATCVKKCPRNYVVTDHGSCVRACGADSYEMEEDGVRKCKKCEGPCRKVCNGIGIGEFKDSLSINATNIKHFKNCTSISGDLHILPVAFRGDSFTHTPPLDPQELDILKTVKEITGFLLIQAWPENRTDLHAFENLEIIRGRTKQHGQFSLAVVSLNITSLGLRSLKEISDGDVIISGNKNLCYANTINWKKLFGTSGQKTKIISNRGENSCKATGQVCHALCSPEGCWGPEPRDCVSCRNVSRGRECVDKCNLLEGEPREFVENSECIQCHPECLPQAMNITCTGRGPDNCIQCAHYIDGPHCVKTCPAGVMGENNTLVWKYADAGHVCHLCHPNCTYGCTGPGLEGCPTNGPKIPSIATGMVGALLLLLVVALGIGLFMRRRHIVRKRTLRRLLQERELVEPLTPSGEAPNQALLRILKETEFKKIKVLGSGAFGTVYKGLWIPEGEKVKIPVAIKELREATSPKANKEILDEAYVMASVDNPH**M**CRLLGICLTSTVQLITQLMPFGCLLDYVREHKDNIGSQYLLNWCVQIAKGMNYLEDRRLVHRDLAARNVLVKTPQHVKITDFGLAKLLGAEEKEYHAEGGKVPIKWMALESILHRIYTHQSDVWSYGVTVWELMTFGSKPYDGIPASEISSILEKGERLPQPPICTIDVYMIMVKCWMIDADSRPKFRELIIEFSKMARDPQRYLVIQGDERMHLPSPTDSNFYRALMDEEDMDDVVDADEYLIPQQGFFSSPSTSRTPLLSSLSATSNNSTVACIDRNGLQSCPIKEDSFLQRYSSDPTGALTEDSIDDTFLPVPEYINQSVPKRPAGSVQNPVYHNQPLNPAPSRDPHYQDPHSTAVGNPEYLNTVQPTCVNSTFDSPAHWAQKGSHQISLDNPDYQQDFFPKEAKPNGIFKGSTAENAEYLRVAPQSSEFIGA

**L861Q**

MRPSGTAGAALLALLAALCPASRALEEKKVCQGTSNKLTQLGTFEDHFLSLQRMFNNCEVVLGNLEITYVQRNYDLSFLKTIQEVAGYVLIALNTVERIPLENLQIIRGNMYYENSYALAVLSNYDANKTGLKELPMRNLQEILHGAVRFSNNPALCNVESIQWRDIVSSDFLSNMSMDFQNHLGSCQKCDPSCPNGSCWGAGEENCQKLTKIICAQQCSGRCRGKSPSDCCHNQCAAGCTGPRESDCLVCRKFRDEATCKDTCPPLMLYNPTTYQMDVNPEGKYSFGATCVKKCPRNYVVTDHGSCVRACGADSYEMEEDGVRKCKKCEGPCRKVCNGIGIGEFKDSLSINATNIKHFKNCTSISGDLHILPVAFRGDSFTHTPPLDPQELDILKTVKEITGFLLIQAWPENRTDLHAFENLEIIRGRTKQHGQFSLAVVSLNITSLGLRSLKEISDGDVIISGNKNLCYANTINWKKLFGTSGQKTKIISNRGENSCKATGQVCHALCSPEGCWGPEPRDCVSCRNVSRGRECVDKCNLLEGEPREFVENSECIQCHPECLPQAMNITCTGRGPDNCIQCAHYIDGPHCVKTCPAGVMGENNTLVWKYADAGHVCHLCHPNCTYGCTGPGLEGCPTNGPKIPSIATGMVGALLLLLVVALGIGLFMRRRHIVRKRTLRRLLQERELVEPLTPSGEAPNQALLRILKETEFKKIKVLGSGAFGTVYKGLWIPEGEKVKIPVAIKELREATSPKANKEILDEAYVMASVDNPHVCRLLGICLTSTVQLITQLMPFGCLLDYVREHKDNIGSQYLLNWCVQIAKGMNYLEDRRLVHRDLAARNVLVKTPQHVKITDFGLAK**Q**LGAEEKEYHAEGGKVPIKWMALESILHRIYTHQSDVWSYGVTVWELMTFGSKPYDGIPASEISSILEKGERLPQPPICTIDVYMIMVKCWMIDADSRPKFRELIIEFSKMARDPQRYLVIQGDERMHLPSPTDSNFYRALMDEEDMDDVVDADEYLIPQQGFFSSPSTSRTPLLSSLSATSNNSTVACIDRNGLQSCPIKEDSFLQRYSSDPTGALTEDSIDDTFLPVPEYINQSVPKRPAGSVQNPVYHNQPLNPAPSRDPHYQDPHSTAVGNPEYLNTVQPTCVNSTFDSPAHWAQKGSHQISLDNPDYQQDFFPKEAKPNGIFKGSTAENAEYLRVAPQSSEFIGA

**x**
