# Supplementary figures and images for: Identification of potential targets of the curcumin analog CCA-1.1 for glioblastoma treatment : integrated computational analysis and in vitro study
Source: Sci Rep. 2022 Aug 17;12:13928. doi: 10.1038/s41598-022-18348-9 (PMC9385707; doi:10.1038/s41598-022-18348-9)

## Slide 1
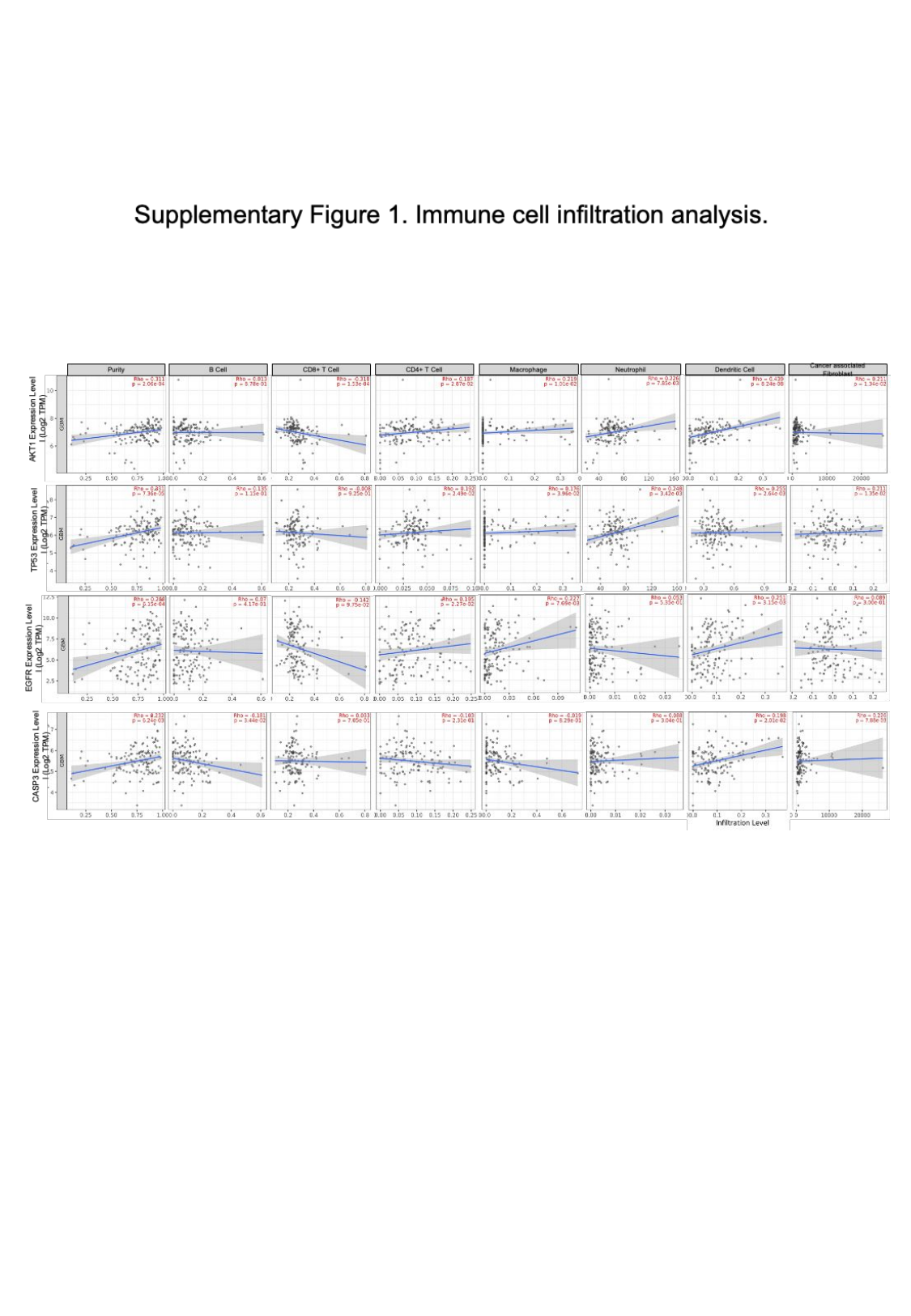

## Slide 2
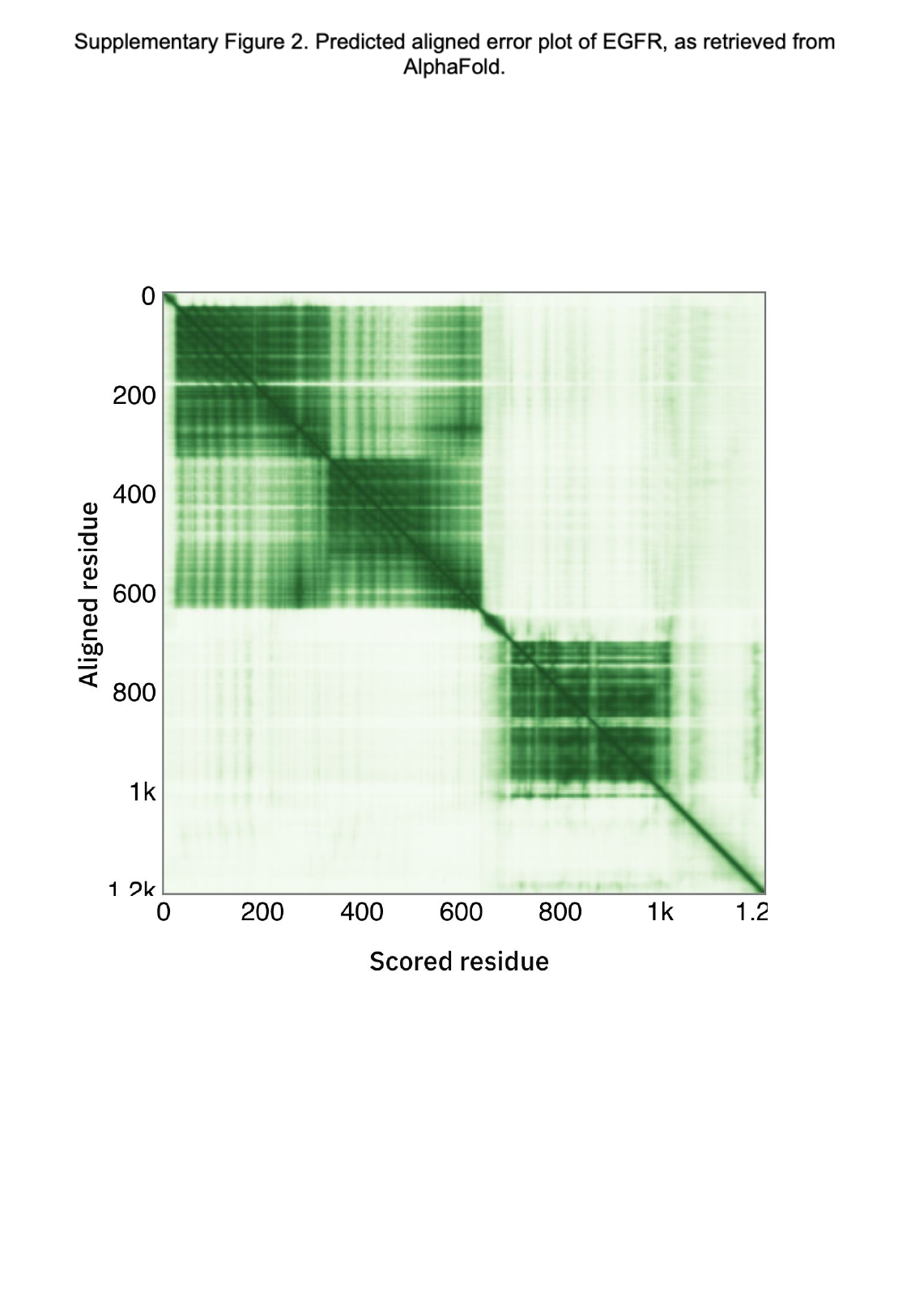

Supplement: Supplementary file 2 — Supplementary Information 2. [file 41598_2022_18348_MOESM2_ESM.pptx]
